# A machine learning model integrating gut microbiota biomarkers for predicting neurological recovery after cerebral hypoxia-ischemia: a single-center study augmented with public data

**DOI:** 10.3389/fphar.2026.1756017

**Published:** 2026-03-31

**Authors:** Ling Kong, Chunhua Wang

**Affiliations:** Department of Pediatric Critical Care Rehabilitation, Guangdong ChunAi Rehabilitation Hospital, Guangzhou, Guangdong, China

**Keywords:** cerebral hypoxia-ischemia, gut microbiome, machine learning, neurological recovery, predictive modeling

## Abstract

**Objective:**

To evaluate the predictive value of gut microbiota biomarkers for neurological recovery after cerebral hypoxia-ischemia and to develop a clinically oriented, validated machine learning (ML) for early outcome prediction.

**Methods:**

In this single-center cohort study, 772 patients (2022–2024) were stratified according to 1-year neurological outcomes into favorable (n = 538) and poor recovery (n = 234) groups, gut microbiota composition, microbial metabolites, systemic inflammatory and oxidative stress markers, and selected gene expression profiles were assessed using blood and fecal samples. Microbial profiling was assessed via 16S rRNA sequencing and whole-genome sequencing. LASSO regression, random forest (RF), and neural networks models were constructed and evaluated using internal validation strategies and augmented with publicly available datasets.

**Results:**

Patients with favorable neurological recovery exhibited significantly higher levels of short-chain fatty acids, increased superoxide dismutase activity, and upregulated neuroprotective gene expression, along with reduced pro-inflammatory cytokines and harmful metabolites (all *P* < 0.001). Microbiota analysis demonstrated enrichment of beneficial taxa (e.g., *Bifidobacterium longum*) and depletion of pro-inflammatory species (e.g., *Clostridium difficile*). Across ML approaches, IL-6 consistently emerged as a key predictive feature, with RF confirmed its prelative importance. The neural network model showed stable predictive performance across validation analyses, indicating robustness for high-dimensional biomarker integration.

**Conclusion:**

Gut microbiota–associated biomarkers, when combined with systemic inflammatory indicators such as IL-6, enable robust early prediction of neurological recovery following cerebral hypoxia–ischemia. ML-based integration of multi-modal biomarkers may facilitate clinically applicable prognostic assessment, while also providing a foundation for future investigations into microbiota-targeted interventions.

## 1 Introduction

Cerebral hypoxia-ischemia represents a frequent emergency within the field of neurocritical care, encompassing various conditions such as ischemic stroke, post-cardiac arrest brain injury, and hypoxic-ischemic encephalopathy in newborns. Its characteristics of high disability and recurrence rates impose a devastating burden on both affected families and the healthcare system (GBD 2019 Stroke Collaborators, 2021; Sekhon et al., 2017). The early and accurate prediction of neurological recovery outcomes is a fundamental prerequisite for effective clinical intervention. Identifying patients at high risk of poor recovery in the initial disease stages would enable the optimization of treatment strategies, such as personalized rehabilitation training, anti-inflammatory therapies, and gut microbiome modulation, thereby significantly improving patient prognosis (Winstein et al., 2016; Chamorro et al., 2012; Singh et al., 2016). However, conventional assessment tools, like the National Institutes of Health Stroke Scale (NIHSS) and the modified Rankin Scale (mRS), primarily rely on subjective symptom scoring (Warner et al., 2019). These measures are considerably influenced by clinician experience and the timing of assessment, and they often fail to detect underlying pathophysiological changes in the early phases of disease. Consequently, their predictive sensitivity and specificity remain suboptimal.

In recent years, the discovery of the gut microbiota-brain axis has provided a novel perspective on the pathophysiology and outcome prediction of cerebral hypoxia-ischemia (Battaglini et al., 2020). The gut microbiota modulates central nervous system function through three principal pathways: Firstly, microbial metabolites, including short-chain fatty acids (SCFAs), trimethylamine N-oxide (TMAO), and indoxyl sulfate, cross the blood-brain barrier via systemic circulation, directly regulating neuronal apoptosis, synaptic plasticity, and neuroinflammation (Lavelle and Sokol, 2020). Secondly, the microbiota activates systemic inflammatory cascades through intestinal mucosal immunity, promoting the release of pro-inflammatory cytokines such as interleukin-6 (IL-6) and tumor necrosis factor-alpha (TNF-α), which exacerbates secondary inflammatory damage following cerebral ischemia (Jiang et al., 2023). Thirdly, gut microbes communicate directly with the central nervous system via neural pathways, including the vagus nerve and the enteric nervous system, thereby participating in neural repair processes (Carlessi et al., 2021). Accumulating evidence confirms significant gut dysbiosis in patients with cerebral hypoxia-ischemia (Wang et al., 2024). Those with favorable recovery outcomes exhibit markedly increased abundances of beneficial bacteria, such as *Bifidobacterium* and *Lactobacillus*, whereas patients with poor recovery show elevated levels of pro-inflammatory bacteria like *Escherichia coli* and *Clostridium difficile*, along with their harmful metabolites (Liang et al., 2025). These findings suggest that gut microbiota and their associated metabolic and inflammatory mediators may serve as potential biomarkers for predicting neurological recovery. Nonetheless, most current studies are confined to small-scale, single-center correlational analyses, lacking large-sample validation and clinically translatable predictive models.

The rapid advancement of machine learning (ML) technology offers a crucial tool for mining high-dimensional biological data. Unlike traditional statistical methods, ML techniques, such as least absolute shrinkage and selection operator (LASSO) regression, random forests (RF), and neural networks, can effectively handle the high-dimensional and sparse nature of gut microbiota data, where only a few out of hundreds of species abundances are relevant to the outcome (Hernández Medina et al., 2022). They also facilitate the integration of multimodal data, including microbial, clinical, and metabolomic information, enabling a comprehensive analysis spanning biomarker selection, model construction, and clinical validation (Greener et al., 2022). However, current ML-based microbiota research still faces three major limitations: First, inadequate sample sizes (most studies include fewer than 200 subjects) increase the risk of overfitting and limit model generalizability. Second, the absence of external validation, rarely utilizing public databases like MGnify or Gene Expression Omnibus (GEO) for cross-cohort verification, undermines the reliability of findings. Third, poor model interpretability renders “black-box” predictions difficult to translate into clinically actionable intervention targets (Tourab et al., 2025; Rozera et al., 2025).

To address these gaps, this study aims to: (1) validate the predictive value of gut microbiota biomarkers for neurological recovery outcomes; (2) construct a clinically practical prediction tool integrating microbiota data with machine learning; and (3) identify novel targets for precise intervention in cerebral hypoxia-ischemia, thereby promoting the application of gut microbiome modulation in neurocritical care.

## 2 Materials and methods

### 2.1 Patient characteristics

A total of 772 patients with cerebral hypoxia-ischemia who were treated at our hospital between January 2022 and December 2024 were enrolled in this study. Based on 1-year follow-up outcomes, patients were categorized into a control group (favorable recovery, n = 538) and a study group (poor recovery, n = 234). The sample size was calculated to ensure adequate statistical power using $n_{1}=n_{2}\times k,n_{2}=\frac{{\left(Z_{\alpha /2}\sqrt{kP\left(1-P\right)}+Z_{\beta }\sqrt{P_{1}\left(1-P_{1}\right)+P_{2}\left(1-P_{2}\right)/k}\right)}^{2}}{{k\left(P_{1}-P_{2}\right)}^{2}}$ . Based on previous studies and derived from clinical practice, the following parameters were defined: a significance level (α) of 0.05, a statistical power (1-β) of 0.8, and a group allocation ratio (k) of 2.30.The baseline characteristics of the two groups are presented in Table 1, showing no significant differences in general demographics such as age and sex. Written informed consent was obtained from all patients or their legal guardians. The study protocol was approved by the Ethics Committee of our hospital.

**TABLE 1:** Baseline characteristics of the patient cohorts.

Baseline characteristic	Control (n = 538)	Study (n = 234)	Statistic	*P*
Age (year)	​	​	χ^2^ = 0.048	0.976
Minor (<18)	285 (53.0%)	125 (53.4%)	​	​
Adult (18–60)	183 (34.0%)	78 (33.3%)	​	​
Elderly (>60)	70 (13.0%)	31 (13.3%)	​	​
Sex	​	​	χ^2^ = 0.065	0.799
Male	312 (58.0%)	138 (59.0%)	​	​
Female	226 (42.0%)	96 (41.0%)	​	​
Residence	​	​	χ^2^ = 0.028	0.866
Urban	362 (67.3%)	156 (66.7%)	​	​
Rural	176 (32.7%)	78 (33.3%)	​	​
Regular diet	485 (90.2%)	198 (84.6%)	χ^2^ = 6.321	0.012
Primary caregiver	492 (91.5%)	195 (83.3%)	χ^2^ = 10.254	0.001
Cerebral hypoxia-ischemia subtype	​	​	χ^2^ = 0.783	0.855
Spastic	395 (73.4%)	175 (74.8%)	​	​
Dyskinetic	68 (12.6%)	25 (10.7%)	​	​
Ataxic	42 (7.8%)	19 (8.1%)	​	​
Mixed	33 (6.1%)	15 (6.4%)	​	​
Comorbid intellectual disability	132 (24.5%)	95 (40.6%)	χ^2^ = 22.865	<0.001
Comorbid language disorder	165 (30.7%)	102 (43.6%)	χ^2^ = 14.923	<0.001
Comorbid epilepsy	52 (9.7%)	41 (17.5%)	χ^2^ = 11.357	0.001
Comorbid visual/hearing impairment	35 (6.5%)	32 (13.7%)	χ^2^ = 9.872	0.002
Comorbid dysphagia	48 (8.9%)	68 (29.1%)	χ^2^ = 45.681	<0.001
GMFCS level (minors)	​	​	Z = 16.325	<0.001
- Level I-II	228 (80.0)	52 (41.6)	​	​
- Level III-V	57 (20.0)	73 (58.4)	​	​
FIM score (adults/elderly)	​	​	Z = 18.957	<0.001
≥60 (moderate/severe independence)	215 (82.1)	79 (47.9)	​	​
<60 (severe dependence)	46 (17.9)	87 (52.1)	​	​
Time from diagnosis to intervention (days)	6.5 ± 3.2	11.2 ± 4.8	t = 16.987	<0.001
Rehabilitation training (≥5 sessions/week)	492 (91.5%)	165 (70.5%)	χ^2^ = 48.752	<0.001
Hypertension	32 (5.9%)	28 (12.0%)	χ^2^ = 8.652	0.003
Diabetes mellitus	21 (3.9%)	25 (10.7%)	χ^2^ = 14.325	<0.001
Coronary heart disease	15 (2.8%)	19 (8.1%)	χ^2^ = 9.768	0.002

GMFCS, gross motor function classification system; FIM, functional independence measure.

Inclusion criteria: (1) Diagnosis of hypoxic-ischemic brain injury (HIBI) by a clear history of an acute hypoxic and/or ischemic event (e.g., cardiac arrest, asphyxia, severe shock, perinatal asphyxia) and be corroborated by at least one of the following objective findings, as confirmed by a neurologist:Cranial computed tomography (CT) or magnetic resonance imaging (MRI) demonstrating abnormalities consistent with hypoxic-ischemic encephalopathy, such as diffuse cerebral edema or watershed zones. Electroencephalography (EEG) revealing severe abnormalities characteristic of hypoxic encephalopathy, such as a burst-suppression pattern or electrographic seizures. (2) Ability to complete at least 1 year of follow-up assessments. (3) Agreement to provide fecal samples for gut microbiota analysis, with complete baseline clinical data and follow-up records, and no missing key information. (4) Provision of voluntary written informed consent by the patient or their legal guardian for participation in the study and use of associated data and samples.

Exclusion criteria: (1) Comorbid severe cardiopulmonary failure, end-stage chronic kidney disease, decompensated liver cirrhosis, malignant tumors, or ongoing radiotherapy/chemotherapy; presence of severe infectious diseases or immunodeficiency. (2) History of significant gastrointestinal diseases, such as inflammatory bowel disease in an active phase, intestinal perforation, or obstruction; use of broad-spectrum antibiotics, probiotics, prebiotics, or undergoing fecal microbiota transplantation within 3 months prior to enrollment, which could potentially alter gut microbiota composition. (3) Comorbid genetic metabolic diseases, hereditary ataxia, Parkinson’s disease, Alzheimer’s disease, or other disorders potentially affecting motor function and neurological recovery, making it difficult to attribute outcomes solely to cerebral hypoxia-ischemia. (4) Presence of severe cognitive impairment, psychiatric disorders, or communication barriers preventing cooperation with neurological recovery assessments; loss to follow-up during the study period due to relocation, loss of contact, death, or participation in other concurrent clinical trials, which could impact the intervention and evaluation outcomes of this study; refusal by the patient or legal guardian to participate in fecal sample collection, gut microbiota testing, or follow-up assessments, or withdrawal of informed consent.

### 2.2 Data collection and laboratory assays

#### 2.2.1 Blood sample processing

Peripheral venous blood (8 mL) was collected from patients after an overnight fast. A 3 mL aliquot was transferred to an EDTA-coated tube for complete blood count analysis (BD Vacutainer®, Becton Dickinson, United States). The remaining 5 mL was placed in a procoagulant tube, allowed to clot for 30 min at room temperature, and subsequently centrifuged at 3,000 × g for 15 min. The resulting serum was aliquoted into microtubes (Axygen Scientific, United States) and stored at −80 °C for subsequent batch analysis of inflammatory cytokines, oxidative stress markers, and routine biochemical parameters.

#### 2.2.2 Fecal sample processing and metabolite quantification

Patients were instructed to collect 5–10 g of fresh fecal sample into a sterile container containing a preservative solution (Majorbio®, Shanghai, China). Samples were transported to the laboratory on ice within 2 h of collection and stored at −80 °C until analysis. Concentrations of gut microbiota-derived metabolites, including SCFAs, indoxyl sulfate, kynurenine, and TMAO, were determined using high-performance liquid chromatography (HPLC) (Agilent 1,260 Infinity II system, Agilent Technologies, United States). Frozen fecal samples were thawed, and 1 g was homogenized in 5 mL of phosphate-buffered saline (PBS; pH 7.4; Gibco, Thermo Fisher Scientific, United States) by vortexing for 30 min. The homogenate was centrifuged at 12,000 × g for 20 min at 4 °C. The supernatant was filtered through a 0.22 μm membrane (Millipore, United States). For serum TMAO analysis, 200 μL of serum was directly filtered through a 0.22 μm membrane. Separation was performed on a C18 column (250 mm × 4.6 mm, 5 μm; Agilent ZORBAX Eclipse Plus C18, United States) using a mobile phase of methanol (HPLC grade, Merck, Germany) and 0.1% phosphoric acid (Sigma-Aldrich, United States) in a gradient elution mode, at a flow rate of 1.0 mL/min and a column temperature of 30 °C. Detection wavelengths were set at 210 nm for SCFAs, 260 nm for indoxyl sulfate and kynurenine, and 220 nm for TMAO. Metabolite concentrations were calculated based on standard curves derived from reference standards (Sigma-Aldrich, United States). Calibration curves showed high linearity (*R* ^2^ > 0.99). Assay precision was evaluated by repeated measurements, demonstrating acceptable reproducibility.

#### 2.2.3 Inflammatory cytokine assay

Serum levels of TNF-α, IL-6, and interferon-gamma (IFN-γ) were quantified using commercial enzyme-linked immunosorbent assay (ELISA) kits (Human TNF-α ELISA Kit, Cat. No. DTA00D; Human IL-6 ELISA Kit, Cat. No. D6050; Human IFN-γ ELISA Kit, Cat. No. DIF50C; R&D Systems, Minneapolis, MN, United States), following the manufacturer’s protocols. Absorbance was measured at 450 nm using a microplate reader (BioTek Synergy H1, Winooski, VT, United States), and cytokine concentrations were interpolated from respective standard curves.

#### 2.2.4 Oxidative stress marker

Superoxide dismutase (SOD) activity in serum was assessed using a commercial xanthine oxidase–based assay kit (No. A001-3-2, Nanjing Jiancheng Bioengineering Institute, China). A 50 μL serum sample was mixed with the assay reagent and incubated at 37 °C for 20 min. The absorbance was measured at 550 nm using a microplate spectrophotometer (BioTek Synergy H1, United States). SOD activity (U/mL) was calculated according to the manufacturer’s formula.

#### 2.2.5 Analysis of gene expression

Total RNA was extracted from peripheral blood mononuclear cells (PBMCs) using Trizol reagent (TRIzol™ Reagent, Invitrogen, Thermo Fisher Scientific, United States). RNA purity and integrity were assessed using a NanoDrop 2000 spectrophotometer (Thermo Fisher Scientific, United States) and agarose gel electrophoresis, respectively. Complementary DNA (cDNA) synthesis was performed using a reverse transcription kit (PrimeScript™ RT Reagent Kit, Takara Bio, Japan). Quantitative polymerase chain reaction (qPCR) was performed using a SYBR Green system (TB Green® Premix Ex Taq™, Takara Bio, Japan) in a 20 μL reaction volume containing 2 μL of cDNA, 0.8 μL each of forward and reverse primers, 10 μL of SYBR Green Mix, and 6.4 μL of nuclease-free water. The thermal cycling conditions were: initial pre-denaturation at 95 °C for 30 s, followed by 40 cycles of 95 °C for 5 s and 60 °C for 30 s. A melting curve analysis was performed to confirm amplification specificity. All qPCR reactions were conducted on a real-time PCR system (ABI 7500 Fast Real-Time PCR System, Applied Biosystems, Foster City, CA, United States). The relative mRNA expression levels of target genes (IL6, CHRNA7, TLR4, OCLN, FFAR3, SLC5A8, FMO3, IDO1) were normalized to the housekeeping gene GAPDH and calculated using the 2^−ΔΔCT^ method. A greater than twofold change in relative expression between groups was considered statistically significant. Candidate genes were pre-specified based on prior experimental and clinical evidence linking gut microbiota–derived metabolites, inflammatory signaling pathways, and blood–brain barrier regulation to neuroimmune modulation in hypoxic–ischemic brain injury.

#### 2.2.6 Routine clinical parameters

A complete blood count, including white blood cell count (WBC), hemoglobin (Hb), and platelet count (PLT), was performed using an automated hematology analyzer (Sysmex XN-1000, Sysmex Corporation, Japan). Liver and kidney function tests, as well as lipid profiles, including alanine aminotransferase (ALT), aspartate aminotransferase (AST), total bilirubin (TBIL), albumin (ALB), serum creatinine (Scr), blood urea nitrogen (BUN), fasting blood glucose (FBG), total cholesterol (TC), triglycerides (TG), high-density lipoprotein cholesterol (HDL-C), and low-density lipoprotein cholesterol (LDL-C), were determined using an automated biochemical analyzer (Hitachi 7,600, Hitachi Ltd., Japan), following standardized operational procedures and regular quality control protocols.

#### 2.2.7 Patient grouping based on 1-year follow-up

Patients were stratified into two groups based on their functional recovery status at the 1-year follow-up:

Minor patients (<18 years): Favorable recovery was defined as an improvement of ≥1 level in the Gross Motor Function Classification System (GMFCS) or an increase of ≥15 points in the Peabody Developmental Motor Scales score. Poor recovery was defined as no improvement or worsening in GMFCS level, or an increase of <5 points in the Peabody score.

Adult and elderly patients (≥18 years): Favorable recovery was defined as an increase of ≥20 points in the Functional Independence Measure (FIM) score or an increase of ≥15 points in the modified Barthel Index. Poor recovery was defined as no improvement or a decrease in FIM score, or an increase of <5 points in the modified Barthel Index.

### 2.3 Gut microbiota whole-genome sequencing (WGS)

Fecal gut microbiota WGS was performed using the Illumina NovaSeq 6,000 platform (Illumina, San Diego, CA, United States). For this analysis, three randomly selected samples from each patient group were subjected to sequencing at Majorbio Bio-Pharm Technology Co., Ltd. (Shanghai, China). The detailed procedure was as follows:

Sample preprocessing and nucleic acid extraction: A 0.2 g aliquot of frozen fecal sample was homogenized in sterile PBS (Gibco, Thermo Fisher Scientific, United States), followed by centrifugation at 8,000 × g for 5 min at 4 °C to remove particulate debris. Total genomic DNA was extracted using the cetyltrimethylammonium bromide (CTAB) method. Proteins and RNA were digested by incubation with proteinase K (Sigma-Aldrich, United States) and RNase A (Takara Bio, Japan), respectively. The DNA was subsequently purified via phenol-chloroform extraction and isopropanol precipitation. The resulting DNA pellet was washed with 75% ethanol, air-dried, and finally resuspended in nuclease-free water. DNA purity was assessed using a Nanodrop spectrophotometer (NanoDrop 2000; Thermo Fisher Scientific, United States) (acceptable A260/A280 ratio: 1.8–2.0), and integrity was verified by agarose gel electrophoresis.

Sequencing library construction: Qualified DNA (1 μg) from each sample was sheared into fragments of approximately 350 bp using a Covaris ultrasonicator (Covaris S220, Covaris Inc., United States). These fragments underwent end repair (to generate blunt ends), were adenylated at the 3′ ends, and were then ligated to Illumina standard adapters containing sample-specific index sequences. Fragments without successfully ligated adapters were removed using magnetic bead-based purification. The library fragments were subsequently amplified via PCR for 12 cycles to enrich the final library. The concentration (required ≥2 nM) and fragment size distribution of the resulting double-stranded DNA libraries were assessed using an Agilent 2,100 bioanalyzer.

Sequencing: Qualified libraries from individual samples were pooled in equimolar concentrations, diluted to a final loading concentration of 1.8 pM, and applied to an Illumina NovaSeq 6,000 flow cell. Sequencing was performed using a paired-end (PE150) strategy, generating 2 × 150 bp reads. During the sequencing process, library fragments were immobilized on the flow cell and amplified via bridge amplification to form clusters. Fluorescently labeled dNTPs and DNA polymerase were used in the sequencing-by-synthesis (SBS) reaction to incorporate nucleotides, with fluorescence signals captured in real-time and translated into base calls. Raw sequencing data was output in FASTQ format. A minimum sequencing depth of 30 × was achieved for all samples, ensuring a minimum genome coverage of ≥95%.

Bioinformatic analysis: Raw sequencing data first underwent quality control, including the removal of low-quality reads (sequences with a Q30 score <80%), adapter contaminants, and duplicate sequences. *De novo* assembly of the quality-filtered reads was performed using MEGAHIT software (version 1.2.9) to generate microbial contig sequences. Coding genes were predicted from the assembled contigs using MetaGeneMark (version 3.38), and a non-redundant gene catalog was constructed. These genes were functionally annotated against the Kyoto Encyclopedia of Genes and Genomes (KEGG) and Clusters of Orthologous Groups (COG) databases. Finally, the high-quality sequencing reads were mapped back to the non-redundant gene catalog using Bowtie2 (version 2.4.5) to calculate gene relative abundance. Differential gene abundance between the Control and Study groups was analyzed using the DESeq2 package (version 1.42.0) in R (version 4.4.3), based on the gene count matrix derived from the mapping results. Genes with an adjusted P-value <0.05 and |log2 fold change| >1 were considered significantly differentially abundant. False discovery rate (FDR) correction was performed using the Benjamini–Hochberg method. This comprehensive analysis pipeline yielded core information on gut microbiota composition, gene functions, and metabolic pathways, providing genomic-level data for the subsequent identification of microbial biomarkers. This comprehensive analysis pipeline yielded core information on microbial gene content and functional pathways, providing genomic-level data for subsequent integrative and predictive analyses.

### 2.4 Gut microbiota analysis

Analysis of the gut microbiota was performed using fresh fecal samples collected at the time of patient enrollment to assess gut microbial community structure and taxonomic composition. The core procedure consisted of sample preprocessing, nucleic acid extraction, PCR amplification, and sequencing.

Frozen fecal samples (0.2 g) were thoroughly vortexed in sterile physiological saline and subsequently centrifuged at 8,000 × g for 5 min at 4 °C to remove particulate debris. Total DNA was extracted from the supernatant using the CTAB method. During extraction, proteins and RNA were digested with proteinase K (Sigma-Aldrich, United States) and RNase A (Takara Bio, Japan), respectively. The DNA was further purified by phenol-chloroform extraction and isopropanol precipitation. DNA purity was confirmed using a Nanodrop spectrophotometer (A260/A280 ratio of 1.8–2.0), and integrity was verified by agarose gel electrophoresis.

PCR amplification was then conducted targeting the hypervariable V4-V5 regions of the bacterial 16S rRNA gene, using the specific primers 515F (5′-GTGCCAGCMGCCGCGGTAA-3′) and 907R (5′-CCGTCAATTCMTTTRAGTTT-3′). The 20 μL reaction mixture contained 2 μL of DNA template, 0.8 μL of each forward and reverse primer, and 10 μL of PCR Master Mix (Takara Bio Inc., Japan). The thermal cycling protocol was as follows: initial pre-denaturation at 95 °C for 3 min; 30 cycles of denaturation at 95 °C for 30 s, annealing at 55 °C for 30 s, and extension at 72 °C for 45 s; followed by a final extension at 72 °C for 5 min. The amplification products were verified by 2% agarose gel electrophoresis. Qualified amplicons were subjected to paired-end sequencing (PE300, Illumina, United States) on an Illumina MiSeq platform (Illumina, San Diego, CA, United States). A minimum of 50,000 high-quality sequencing reads were generated per sample to ensure adequate coverage of microbial diversity.

Raw sequencing data in FASTQ format underwent stringent quality control. This process involved discarding low-quality sequences (defined as those with a Q30 score below 80%), adapter contaminants, and duplicate reads. The paired-end reads were merged into full-length 16S rRNA gene fragments using FLASH software (version 1.2.11). These sequences were then clustered into operational taxonomic units (OTUs) at a 97% similarity threshold using the UPARSE algorithm (USEARCH version 11.0), during which singleton OTUs (sequences appearing only once) were removed to minimize potential errors. Taxonomic classification of the OTUs, from phylum to genus level, was performed using the RDP Classifier tool (version 2.13) against the SILVA database (version 138). Due to the inherent resolution limitations of 16S rRNA sequencing and database-dependent annotation, a subset of OTUs could not be confidently assigned to genus- or species-level taxonomy. These OTUs were retained as distinct features in the abundance matrix for statistical modeling but are presented as “Microbe #” in figures where applicable. Biological interpretation in the manuscript focuses primarily on taxa with reliable taxonomic annotation.

### 2.5 Data processing and curation

Distinct criteria were established for handling missing data based on data type. All data preprocessing and curation procedures were performed using R software (version 4.4.3). For clinical baseline data (e.g., age, sex, cerebral hypoxia-ischemia subtype), samples with a missing value rate exceeding 5% were excluded entirely. For variables with a missing rate ≤5%, imputation was performed: categorical variables (e.g., residence, feeding mode) were imputed using the mode (the most frequent value within the group), while continuous variables (e.g., age at diagnosis, GMFCS score) were imputed using the mean of non-missing values within the same group. Laboratory measures (e.g., SCFAs, IL-6) and gene expression data, having undergone stringent quality control during assaying, exhibited minimal missingness primarily due to assay failure; corresponding samples were directly excluded. For gut microbiota sequencing data, samples yielding an effective read count below 30,000 or an OTU annotation rate below 80% were deemed incomplete and were either re-sequenced or excluded from subsequent analysis.

Potential outliers in continuous data (e.g., inflammatory cytokine concentrations, SOD activity) were initially identified using the boxplot method (values exceeding 1.5 times the interquartile range). These were further scrutinized against clinical reference ranges (e.g., IL-6 > 100 pg/mL, far exceeding the pathological upper limit) for validation. Suspected outliers were confirmed through laboratory re-inspection. If a laboratory error was confirmed, the value was replaced with the median of its group. If the extreme value was deemed biologically plausible (e.g., from a patient with a concurrent special infection), it was retained and annotated. For categorical variables (e.g., cerebral hypoxia-ischemia subtype, functional level), logical consistency was verified using cross-tabulation. Contradictory entries, such as an “adult patient assigned a GMFCS level” or a “spastic cerebral hypoxia-ischemia co-labeled as ataxic type”, were corrected by referring to the original medical records; if unresolvable, the sample was excluded. In sequencing data, instances of “extreme abundance values” (OTU relative abundance >10% observed in only a single sample) were considered potential sequencing contaminants and were removed.

Data formatting and units were standardized across the dataset. “Age” in clinical records was uniformly converted to “decimal years” (retaining one decimal for infants). “Rehabilitation training frequency” was standardized to “sessions per week”. Laboratory measurement units were calibrated to international standards (e.g., TMAO concentration was converted from “mg/L” to “μmol/L″). Gene relative expression levels were uniformly normalized to the GAPDH reference gene, ensuring consistent application of the 2^−ΔΔCT^ calculation method across all samples.

The dataset was refined by removing redundancies. Duplicate sample entries were identified and deleted based on a unique “patient ID + test date” combination. Variables irrelevant to the research objectives (e.g., patient history of common cold, non-relevant laboratory parameters) were removed. Synonymous variables (e.g., “regular rehabilitation” and “weekly training ≥5 sessions”) were consolidated into a single standardized variable (“high-intensity rehabilitation”). This rigorous curation process resulted in a clean, well-structured, and standardized dataset, providing a robust foundation for subsequent machine learning modeling and statistical analysis. After data cleaning and curation, all retained variables were classified into predefined feature domains, including (1) demographic and clinical characteristics, (2) laboratory biomarkers and gut microbiota–derived metabolites, (3) gene expression variables derived from PBMCs and WGS analyses, and (4) taxonomically annotated gut microbial features. These curated variables constituted the candidate feature pool for subsequent predictive modeling. Variables excluded during the curation process were not considered in downstream analyses.

### 2.6 Predictive model construction

The binary 1-year recovery outcome (favorable vs. poor) was defined as the dependent variable. Predictive modeling was conducted using the curated multi-modal feature pool described in Section 1.5, including baseline clinical variables, laboratory biomarkers, microbiota-derived metabolites, PBMC gene expression markers, WGS-derived differential genes, and taxonomically annotated microbial abundance features. All predictive models were implemented using R software version 4.5.1.

A LASSO regression model was developed on the training dataset using the glmnet package (version ≥4.1). All candidate features from the curated multi-modal feature pool were initially included as potential predictors. The optimal regularization parameter (λ) was determined via 5-fold cross-validation (5-fold CV), which concurrently selected features with non-zero coefficients. Model performance was evaluated on the test set by calculating accuracy, the area under the receiver operating characteristic curve (AUC-ROC), sensitivity, and specificity. Prediction errors were analyzed using the confusion matrix, and the direction of each feature’s effect on the outcome was interpreted based on the sign (positive or negative) of its coefficient.

A RF classifier was constructed using the predictors retained by the LASSO model (non-zero coefficients), implemented via the randomForest package (version ≥4.7) with the following core parameters: the number of decision trees (n_estimators) was optimized through a grid search (range: 100–1,000, step size: 100), yielding an optimal value of 500; the minimum number of samples required to split an internal node (min_samples_split) was set to 5, and the minimum number of samples required to be at a leaf node (min_samples_leaf) was set to 2. Parameters were fine-tuned using 5-fold cross-validation, with the minimization of the out-of-bag (OOB) error serving as the objective function. The model was assessed on the test set using the same metrics as the LASSO model. Additionally, model stability was verified via permutation testing. The final model provided a feature importance ranking, from which key biomarkers were selected for further consideration based on their clinical relevance.

Feature variables were normalized to a [0, 1] range using Min-Max scaling (implemented via base R functions) prior to neural network modeling. Only variables retained after the combined feature selection procedures (LASSO and RF) were used as inputs to the neural network model, thereby reducing dimensionality and mitigating overfitting. The training set was further split into a sub-training set and a validation set (80:20 ratio), with the latter used to implement early stopping during training. A 3-layer fully connected neural network architecture was employed using the nnet package (version ≥7.3), comprising an input layer, a hidden layer, and an output layer. The network was trained using the Adam optimizer with a learning rate of 0.001 and a cross-entropy loss function. A Dropout layer was incorporated after the hidden layer to mitigate overfitting. Training was configured for a maximum of 50 epochs with an early stopping mechanism enabled. The model’s predictive performance was evaluated on the test set based on accuracy, AUC, and the F1-score. To enhance interpretability, SHapley Additive exPlanations (SHAP) values were computed using the iml package (version ≥0.11) to quantify feature contributions and visualize the directional impact of core features (e.g., IL-6, SCFAs) on the predictions.

### 2.7 Group comparisons and regression analysis

All continuous data were confirmed to be normally distributed. Statistical analyses were performed using SPSS (version 27.0; IBM Corp., Armonk, NY, United States) and R (version 4.4.3; R Foundation for Statistical Computing, Vienna, Austria). Normally distributed continuous data were presented as the mean ± standard deviation ( $\bar{x}$ ± s), while categorical data are summarized as counts and percentages (n, %). Intergroup comparisons for continuous variables were conducted using the independent samples *t*-test, and associations between categorical variables were assessed with the chi-square test. To identify independent risk factors, a multivariable binary logistic regression analysis was employed. Data visualization was performed using the ggplot2 package (version 4.0.1) in R. A two-sided *P*-value <0.05 was considered statistically significant.

## 3 Results

### 3.1 Baseline characteristics

Baseline demographic and clinical characteristics of the Control and Study groups are summarized in Table 1. The two groups were comparable with respect to age distribution, sex, residence, and cerebral hypoxia–ischemia subtype, with no statistically significant differences observed (all P > 0.05). In contrast, several baseline clinical characteristics differed significantly between groups. The Study group showed a higher prevalence of comorbid intellectual disability, language disorder, epilepsy, visual or hearing impairment, and dysphagia compared with the Control group (all P < 0.01). Baseline functional status was also significantly poorer in the Study group, as indicated by higher GMFCS levels among minors and lower FIM scores among adults and elderly patients (both P < 0.001). In addition, the Study group exhibited a longer time from diagnosis to intervention, lower intensity of rehabilitation training, and a higher prevalence of hypertension, diabetes mellitus, and coronary heart disease (all P < 0.01). Regarding data completeness, missing values were limited to a small number of baseline variables, primarily dietary pattern, primary caregiver status, and selected comorbidity indicators. The proportion of missing data per variable ranged from approximately 1%–5%. As predefined in the Methods, these missing values were handled using group-wise imputation. No variable exceeded the prespecified threshold for exclusion.

### 3.2 Comparison of laboratory parameters

Comparative analysis revealed significant differences in gut microbiota-derived metabolites between the control and study groups. The control group demonstrated markedly higher levels of SCFAs (65.2 ± 12.8 vs. 42.8 ± 10.5 mmol/L), whereas concentrations of indoxyl sulfate, kynurenine, and TMAO were significantly elevated in the study group (all *P* < 0.001). These findings indicate distinct microbial metabolic profiles between patients with favorable and poor recovery. A pronounced systemic inflammatory state was evident in the study group, which exhibited significantly higher serum levels of TNF-α, IL-6, and IFN-γ compared to the control group (all *P* < 0.001). Gene expression analysis further delineated the molecular disparities. The expression levels of genes associated with neuroprotection, CHRNA7, OCLN, FFAR3, and SLC5A8, were significantly higher in the control group. Conversely, the study group showed upregulation of genes linked to pro-inflammatory responses and harmful metabolite processing, including IL6, TLR4, FMO3, and IDO1 (all *P* < 0.001). Antioxidant capacity was also compromised in the study group, as indicated by significantly lower serum SOD activity compared to the control group (128.5 ± 15.6 vs. 86.3 ± 12.8 U/mL, *P* < 0.001). In contrast, comparisons of routine clinical parameters, including liver enzymes (ALT, AST), TBIL, renal function markers (Scr, BUN), lipid profiles (TC, TG, HDL-C, LDL-C), and complete blood count components (WBC, Hb, PLT), showed no statistically significant differences (all *P* > 0.05). Minor differences were observed in ALB and FBG levels; however, the absolute values for both fell within normal reference ranges, rendering these differences clinically insignificant. The overall comparability of these routine parameters strengthens the assertion that the observed associations between the target biomarkers (gut microbiota markers, inflammatory cytokines, and related genes) and recovery outcomes are specific and not confounded by baseline differences in hepatic, renal, metabolic, or hematologic function (Table 2).

**TABLE 2:** Comparative analysis of laboratory parameters between patient groups.

Indicator	Control group (n = 538)	Study group (n = 234)	Statistic	*P*
SCFAs (mmol/L)	65.2 ± 12.8	42.8 ± 10.5	t = 28.653	<0.001
Indoxyl sulfate (μmol/L)	8.5 ± 2.3	15.7 ± 3.6	t = 26.987	<0.001
Kynurenine (μmol/L)	1.2 ± 0.4	2.8 ± 0.7	t = 31.254	<0.001
TMAO (μmol/L)	4.8 ± 1.5	10.3 ± 2.8	t = 27.362	<0.001
TNF-α (pg/mL)	12.5 ± 3.2	28.7 ± 5.6	t = 35.871	<0.001
IL-6 (pg/mL)	8.6 ± 2.1	21.4 ± 4.3	t = 38.925	<0.001
CHRNA7	2.5 ± 0.6	1.1 ± 0.4	t = 34.723	<0.001
TLR4	1.2 ± 0.3	2.9 ± 0.7	t = 30.895	<0.001
OCLN	3.1 ± 0.8	1.5 ± 0.5	t = 29.632	<0.001
FFAR3	2.8 ± 0.7	1.2 ± 0.4	t = 33.571	<0.001
SLC5A8	2.6 ± 0.6	1.0 ± 0.3	t = 36.285	<0.001
FMO3	1.1 ± 0.3	2.7 ± 0.6	t = 31.954	<0.001
IDO1	1.0 ± 0.2	2.5 ± 0.5	t = 38.712	<0.001
SOD (U/mL)	128.5 ± 15.6	86.3 ± 12.8	t = 37.526	<0.001
ALT (U/L)	25.3 ± 8.6	24.8 ± 7.9	t = 0.892	0.373
AST (U/L)	28.5 ± 9.2	27.9 ± 8.7	t = 0.956	0.34
TBIL (μmol/L)	12.8 ± 4.3	13.1 ± 4.1	t = 0.873	0.383
ALB (g/L)	42.5 ± 3.8	41.9 ± 3.6	t = 1.987	0.048
Scr (μmol/L)	68.5 ± 12.3	70.2 ± 11.8	t = 1.765	0.078
BUN (mmol/L)	5.2 ± 1.3	5.4 ± 1.2	t = 1.892	0.059
FBG (mmol/L)	5.4 ± 0.8	5.6 ± 0.9	t = 2.154	0.032
TC (mmol/L)	4.8 ± 0.7	4.9 ± 0.8	t = 1.632	0.103
TG (mmol/L)	1.5 ± 0.6	1.6 ± 0.5	t = 1.956	0.051
HDL-C (mmol/L)	1.4 ± 0.3	1.3 ± 0.4	t = 1.789	0.074
LDL-C (mmol/L)	2.8 ± 0.6	2.9 ± 0.7	t = 1.562	0.119
WBC (×10^9^/L)	6.8 ± 1.5	7.0 ± 1.6	t = 1.453	0.147
Hb (g/L)	135.2 ± 12.8	133.9 ± 13.2	t = 1.287	0.2
PLT (×10^9^/L)	235.6 ± 45.8	241.3 ± 42.5	t = 1.592	0.112

SCFAs, short-chain fatty acids; TMAO, trimethylamine N-oxide; TNF-α, tumor necrosis factor-alpha; IL-6, interleukin-6; SOD, superoxide dismutase; ALT, alanine aminotransferase; AST, aspartate aminotransferase; TBIL, total bilirubin; ALB, albumin; Scr, serum creatinine; BUN, blood urea nitrogen; FBG, fasting blood glucose; TC, total cholesterol; TG, triglycerides; HDL-C, high-density lipoprotein cholesterol; LDL-C, low-density lipoprotein cholesterol; WBC, white blood cell count; Hb, hemoglobin; PLT, platelet count.

### 3.3 Differential gene analysis from WGS

**FIGURE 1:**
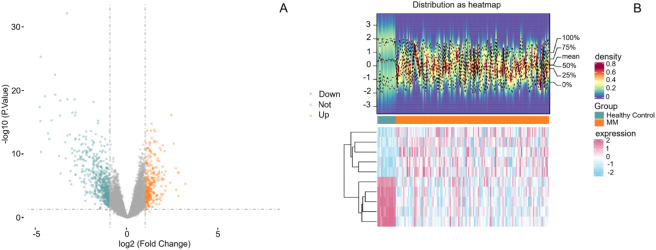
Differential gene abundance analysis based on WGS. **(A)** Volcano plot illustrating the distribution of differentially expressed genes between the two groups. Genes meeting the predefined significance criteria are shown, highlighting the overall extent and direction of transcriptional alterations. **(B)** Heatmap of differentially expressed genes across samples, illustrating the consistency of expression patterns within and between groups.

**FIGURE 2:**
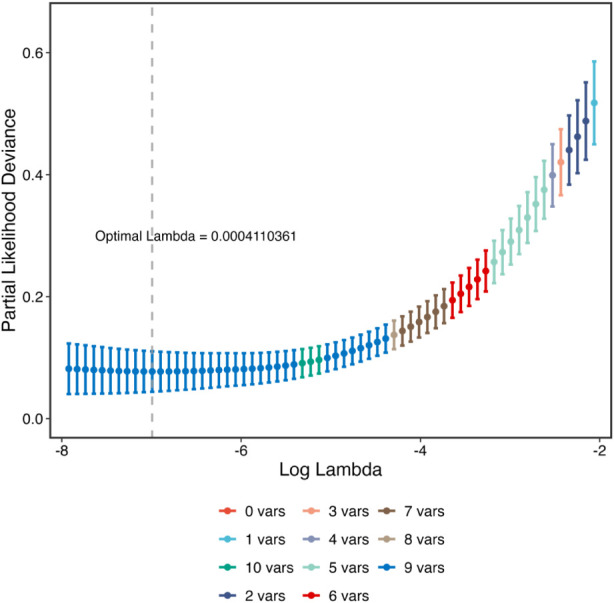
Feature selection of differential genes via LASSO regression. LASSO regression was applied to the differentially expressed genes to perform feature selection. Genes with non-zero coefficients at the optimal penalty parameter were retained as key features associated with the clinical phenotype.

Differential gene abundance analysis based on whole-genome sequencing identified 379 genes with significantly different relative abundance between the two groups, including 213 upregulated and 166 downregulated genes (Figure 1). These results demonstrate marked differences in microbial functional gene composition between clinical phenotypes. LASSO regression was subsequently applied to the differentially abundant genes to identify key features (Figure 2). A parsimonious panel of genes was retained, including SLC5A8, FMO3, IDO1, TNF, IL6, CHRNA7, TLR4, OCLN, and FFAR3.

### 3.4 16S rRNA-based gut microbiota taxonomic analysis

**FIGURE 3:**
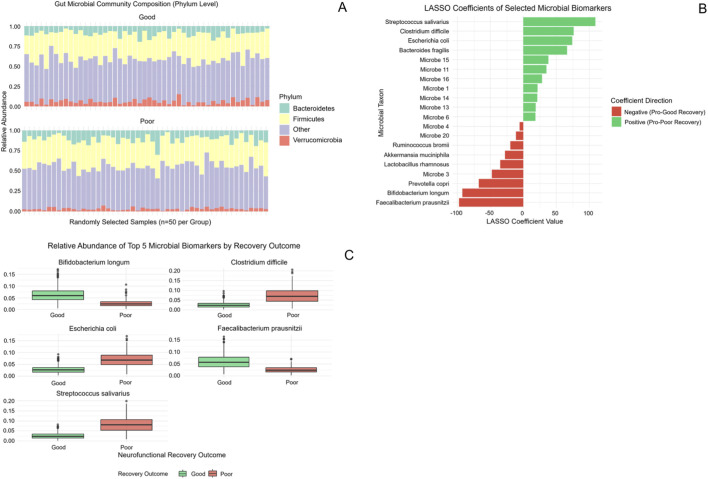
Gut microbiota composition and recovery-associated microbial features. **(A)** Phylum-level gut microbial composition in patients with good and poor neurological recovery, demonstrating broadly similar community structure between groups. **(B)** LASSO coefficient of selected microbial taxa, indicating the direction and magnitude of their association with recovery outcome. **(C)** Relative abundances of the top five taxonomically annotated microbial biomarkers stratified by neurological recovery outcome.

Taxonomic profiling based on 16S rRNA sequencing showed broadly comparable phylum-level community structure between patients, with *Bacteroidetes* and *Firmicutes* remaining dominant (Figure 3A). LASSO regression applied to microbial abundance data identified recovery-associated taxa (Figure 3B). Among annotated taxa, *Bifidobacterium longum* and Faecalibacterium prausnitzii were enriched in patients with favorable recovery, whereas *Clostridium difficile*, *Escherichia coli*, and *Streptococcus salivarius* were more abundant in the poor recovery group (Figure 3C). These taxa-specific differences indicate that recovery status is associated with discrete microbial signatures rather than global compositional shifts.

### 3.5 Identification of significant predictors via LASSO regression

**FIGURE 4:**
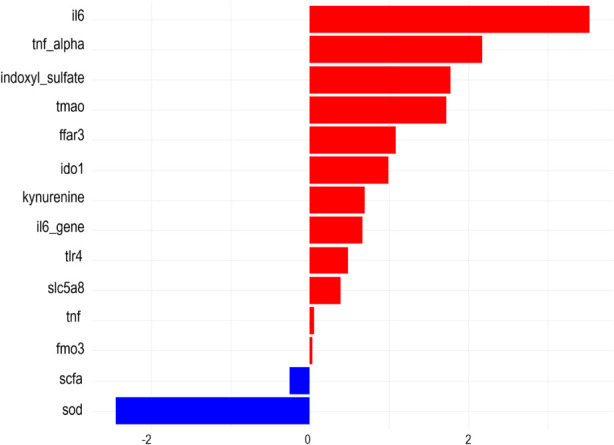
Feature selection using LASSO regression. Regression coefficients of variables retained by the LASSO model, illustrating the direction and relative magnitude of their associations with the study outcome.

LASSO regression was applied for variable selection. The analysis retained a parsimonious set of predictors with non-zero coefficients (Figure 4). IL-6 exhibited the largest positive coefficient, indicating the strongest association with poor recovery. Several additional inflammatory and metabolic variables were also positively associated with outcome. In contrast, SOD demonstrated a negative coefficients, indicating an inverse association with adverse recovery.

### 3.6 Random forest analysis and model comparison

**FIGURE 5:**
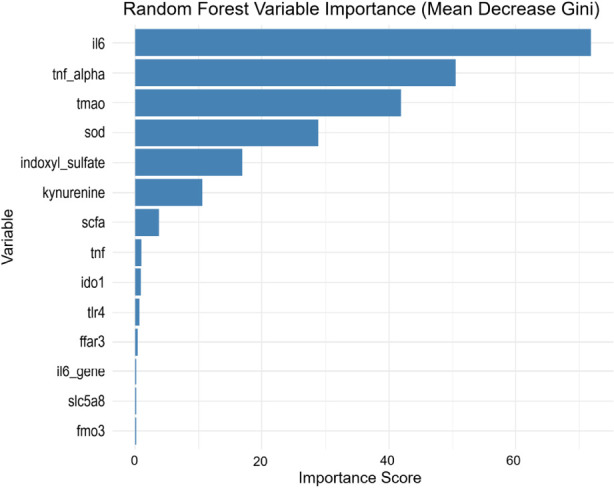
Random forest analysis. A Variable importance ranking derived from the random forest model, highlighting predictors contributing most to outcome discrimination.

RF analysis was performed to further evaluate the relative importance of predictors. Consistent with the LASSO results, IL-6 ranked as the most important variable, exhibiting the highest importance score among all predictors (Figure 5). TNF-α and TMAO also contributed substantially to classification performance, whereas several other variables showed comparatively lower importance. To assess whether a reduced predictor set could maintain performance, an RF model incorporating the top-ranked variables was constructed. The reduced model achieved discrimination comparable to that of the full LASSO-selected model, indicating that the primary predictive signal was concentrated in a limited number of key variables.

### 3.7 Neural network model performance

**FIGURE 6:**
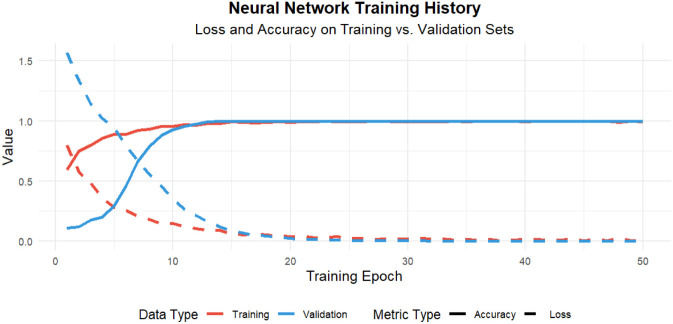
Neural network model performance. Training history of the neural network model demonstrating stable performance across training and validation datasets.

A neural network was trained to further evaluate the predictive capacity of the selected variables. The model demonstrated consistent predictive performance on both the training and validation datasets, supporting its generalization capability (Figure 6). Overall discrimination performance remained stable across datasets, indicating that the selected predictors provided robust and reproducible predictive information.

## 4 Discussion

This study, utilizing a cohort of 772 cerebral hypoxia-ischemia patients spanning all age groups, validates the value of gut microbiota biomarkers combined with ML in predicting neurological recovery outcomes through the analysis of laboratory parameters, gut microbial features, and ML model performance. In this multi-age cohort, poor neurological recovery was characterized by a coordinated pattern of altered gut microbial metabolites, heightened systemic inflammation, impaired antioxidant capacity, and differential expression of neuro-related genes. These biological alterations were consistently identified through multi-omics profiling and further supported by machine learning-based feature selection, suggesting the presence of a convergent microbiota–inflammation–neuroimmune axis potentially associated with recovery heterogeneity.

Our investigation revealed marked disparities in gut microbiota-derived metabolites, inflammatory cytokines, oxidative stress markers, and neuro-related gene expression between patients with poor recovery (study group) and those with favorable recovery (control group). Together, these alterations are consistent with coordinated dysregulation of the microbiota–inflammation–neuroimmune regulatory axis in patients with poor recovery. Regarding metabolic markers, the control group exhibited significantly higher levels of SCFAs, alongside lower concentrations of indoxyl sulfate, kynurenine, and TMAO. Clinical studies in acute ischemic stroke patients have reported that higher levels of certain plasma SCFAs, such as isovalerate, are associated with milder neurological deficits and more favorable functional outcomes following recanalization therapy, supporting the clinical relevance of SCFA alterations observed in our cohort (Chou et al., 2023). This metabolic profile is consistent with prior evidence linking SCFAs to anti-inflammatory and neuroprotective effects within the gut–brain axis. SCFAs, core metabolites of beneficial gut bacteria, confer neuroprotection and exert anti-inflammatory effects by crossing the blood-brain barrier and modulating microglial activation (Socała et al., 2021). Conversely, TMAO exacerbates cerebral inflammation through activation of the Toll-like receptor 4 (TLR4) pathway, while indoxyl sulfate promotes oxidative stress–related neural injury (Adesso et al., 2017; Wang et al., 2025). Elevated circulating TMAO has been associated with increased ischemic stroke severity and unfavorable functional outcomes in clinical cohorts, indicating that higher TMAO levels may correlate with poorer neurological repair in human cerebrovascular injury (Li et al., 2024). The elevation of these metabolites in the poor recovery group therefore provides biological plausibility for their association with impaired neurological repair.

Inflammatory and oxidative stress markers were also markedly altered in patients with poor recovery. The study group demonstrated significantly elevated serum levels of TNF-α and IL-6, coupled with reduced SOD activity. Elevated IL-6 and TNF-α levels have been associated with unfavorable neurological outcomes in ischemic stroke patients, supporting the clinical relevance of systemic inflammation in human cerebrovascular injury (Ciancarelli et al., 2022). IL-6, a pivotal pro-inflammatory cytokine, promotes post-injury inflammatory signaling through JAK-STAT pathway activation and has been associated with impaired neural repair processes (Erta et al., 2012). The concomitant reduction in SOD activity suggests weakened antioxidant defense capacity, potentially facilitating reactive oxygen species–mediated neuronal damage (Sienes Bailo et al., 2022). The coexistence of heightened inflammatory signaling and diminished antioxidant protection supports a pathogenic framework in which inflammation–oxidative stress imbalance contributes to unfavorable neurological outcomes (Cobley et al., 2018).

At the gene expression level, the control group exhibited higher expression of genes implicated in neuroimmune regulation and barrier integrity (CHRNA7, OCLN, FFAR3), whereas pro-inflammatory and metabolite-related genes was upregulated in the poor recovery group. Elevated CHRNA7 (encoding the α7 subunit of the nicotinic acetylcholine receptor) expression is consistent with enhanced cholinergic anti-inflammatory pathway, which may attenuate peripheral-to-central inflammatory transmission (Han et al., 2017). Reduced CHRNA7 expression has also been reported in experimental and clinical studies of neuroinflammatory conditions, where diminished cholinergic anti-inflammatory signaling was associated with worsened neurological outcomes (Prowle et al., 2015). Increased OCLN (occludin) expression suggests better preservation of blood-brain barrier integrity, thereby limiting inflammatory infiltration (Kim et al., 2020). Conversely, FMO3 (flavin-containing monooxygenase 3), a key enzyme involved in TMAO synthesis, was more highly expressed in the poor recovery group, potentially contributing to increased production of pro-inflammatory metabolites (Zhou et al., 2024). Collectively, these transcriptional patterns suggest that gut microbiota–associated metabolic alterations may be accompanied by coordinated host immune and barrier-related gene responses detectable in peripheral blood.

Employing both 16S rRNA sequencing and WGS, our study identified recovery-associated alterations in gut microbial composition and functional gene profiles. At the community structure level, relative abundances of *Bacteroidetes* and *Firmicutes* differed between groups, suggesting compositional shifts that may accompany recovery heterogeneity. Reduced *Bacteroidetes* abundance in the poor recovery group corresponded with elevated harmful metabolites, whereas enrichment of pro-inflammatory taxa within the *Firmicutes* (e.g., *Clostridium* species) may contribute to heightened inflammatory signaling (Zang et al., 2023). At the genus level, *Bifidobacterium longum* was more abundant in patients with favorable group. As an SCFA-producing gut bacterium, it has been associated with improved gut barrier integrity and attenuation of neuroinflammation in experimental models (Lee et al., 2020). In contrast, *Clostridium difficile* was enriched in the poor recovery group; its toxins A/B are known to disrupt mucosal integrity and promote systemic inflammation (Costa et al., 2021). The functional relevance and detectability of these taxa highlight their potential value as non-invasive indicators of recovery-associated microbial states (Yao et al., 2021). Importantly, consistent microbial disparities were observed across pediatric, adult, and elderly subgroups, indicating that these associations were not restricted to a specific age category.

LASSO regression, with 5-fold cross-validation identified IL-6, SOD, TMAO, and *B. longum* abundance as key predictors of recovery. These selected variables were biologically coherent and consistently associated with inflammatory and microbial pathways implicated in recovery outcomes. Although several of these biomarkers have been individually reported in prior studies, the present analysis integrates them within a unified predictive framework, allowing simultaneous evaluation of microbial, metabolic, and inflammatory contributions to recovery (Xie et al., 2024). Random forest analysis further confirmed IL-6 as the most influential predictor. Importantly, a reduced model incorporating top-ranked predictors achieved discrimination comparable to the full model, indicating that a limited set of biomarkers captured most of the predictive signal. This finding suggests that clinically feasible risk stratification could potentially be implemented using a focused biomarker panel, thereby improving practicality in routine hospital settings. The neural network model maintained stable predictive performance across datasets, supporting the robustness of the selected feature set within this cohort. By combining multi-omics biomarkers into a single interpretable framework, the model offers a structured and potentially scalable approach for early identification of patients at higher risk of poor neurological recovery. Furthermore, SHAP analysis was applied to quantify feature contributions. This approach enhanced model transparency by identifying the relative contribution of individual biomarkers to prediction outputs, thereby facilitating clinical interpretation rather than functioning as a purely technical prediction tool.

Several limitations should be considered when interpreting these findings. First, the retrospective cohort design remains susceptible to information bias despite predefined inclusion and exclusion criteria. Although both 16S rRNA sequencing and WGS were performed, integrated metabolomicprofiling was not available, limiting direct assessment of host–microbial metabolic interactions. The WGS analysis was conducted in a limited subset of samples (n = 3 per group), restricting statistical power for detecting differentially abundant genes and increasing the possibility of false-positive findings. Accordingly, the WGS-derived gene signatures should be regarded as exploratory and require validation in larger, independent cohorts. Methodological constraints inherent to gut microbiome research should also be acknowledged. Factors such as sample handling intervals, microbial cell lysis efficiency, and freeze–thaw effects may influence relative abundance estimates. In particular, variations in lysis approaches, including the absence or variability of mechanical disruption (e.g., bead-beating), may lead to differential extraction efficiency across bacterial taxa, potentially biasing detection of certain Gram-positive organisms. Although standardized procedures were applied, residual technical variability cannot be fully excluded. In addition, gene expression profiling was limited to peripheral blood mononuclear cells; the absence of central nervous system tissue precludes direct confirmation of microbiota-associated gene regulation within the brain. External validation of the predictive models is currently limited. While internal cross-validation was implemented, model performance across independent healthcare systems and diverse patient populations remains to be established. Finally, the 1-year follow-up period permits evaluation of short-term neurological recovery only; the prognostic value of gut microbiota biomarkers for long-term outcomes requires further longitudinal investigation.

## 5 Conclusion

In this cohort of patients with cerebral hypoxia-ischemia, gut microbiota–associated metabolites, inflammatory markers, and related gene expression profiles were significantly associated with neurological recovery outcomes. Predictive models incorporating these biologically relevant indicators demonstrated stable discrimination performance, highlighting their potential utility in outcome stratification. Although external validation and mechanistic studies are warranted, these findings contribute to a growing body of evidence linking the gut microbiota–inflammation axis to neurological recovery and support further investigation into microbiota-informed prognostic strategies.

## Data Availability

Due to patient privacy and institutional regulations, the datasets generated during the current study are not publicly available but are available from the corresponding author upon reasonable request.
